# A Stepwise Approach to Managing Bilateral Maxillary Sinus Tracts: A Case Report

**DOI:** 10.1002/ccr3.73037

**Published:** 2026-06-25

**Authors:** Ali Chamani, Maryam Forghani, Reza Shakiba, Arsalan Shahri

**Affiliations:** ^1^ Department of Endodontics, School of Dentistry Mashhad University of Medical Sciences Mashhad Iran; ^2^ Student Research Committee, School of Dentistry Mashhad University of Medical Sciences Mashhad Iran; ^3^ Dental Materials Research Center, Mashhad Dental School Mashhad University of Medical Sciences Mashhad Iran

**Keywords:** dental fistula, endodontics, focal infection, root canal therapy, tooth fractures

## Abstract

This case report describes a rare presentation of bilateral intraoral sinus tracts of odontogenic origin associated with a maxillary right first molar (Universal #3). A key diagnostic challenge was differentiating an endodontic infection from vertical root fracture (VRF) in a previously treated tooth; no J‐shaped probing defect was detected. The tooth was managed with single‐visit nonsurgical root canal retreatment, resulting in symptom resolution and progressive radiographic healing. At nine‐month follow‐up, both sinus tracts had completely resolved, and the tooth remained asymptomatic and functional.

## Key Clinical Message

Bilateral maxillary sinus tracts may originate from a single endodontic lesion without vertical root fracture. Careful dental‐first evaluation, including periodontal probing and radiographic assessment, can support a conservative diagnosis. Nonsurgical root canal retreatment with adequate follow‐up can resolve tracts, preserve the tooth, and avoid unnecessary surgical or extraction procedures altogether.

## 1 Introduction

A longstanding odontogenic infection can lead to pus formation, which drains intra‐orally or extra‐orally through sinus tracts. Sinus tracts can appear in various anatomical regions and have sometimes been mistaken for epidermoid cysts, cystic acne, or salivary gland fistulas [1]. There have been reports of cutaneous fistulas (both unilateral and bilateral) caused by dental origins [2, 3]. Additionally, many nasal sinus tracts have been documented [4, 5, 6]. Accordingly, atypical sinus tract presentations warrant a high index of suspicion for an odontogenic origin to minimize misdiagnosis.

Lesions in atypical locations can lead to misdiagnosis, unnecessary treatments, and persistent symptoms or complications [7]. Given the limited literature on this topic, each case report adds valuable insight. This report describes a case of bilateral sinus tracts in a young woman and reviews the relevant literature to provide a better understanding of their causes and management. To our knowledge, such presentations are rarely described; following a literature search, we found no previous reports of an alveolar bilateral sinus tract linked to a maxillary first molar.

## 2 Case Presentation

This case report study is documented based on PRICE 2020 guidelines [8]. Additionally, all procedures complied with the Declaration of Helsinki. An endodontic specialist with 5 years of experience performed all treatment protocols in the endodontics department of Mashhad Dental Faculty. Written and verbal informed consent was obtained from the patient. The patient also consented to publication of this case and accompanying images.

### 2.1 Case History and Examination

**FIGURE 1:**
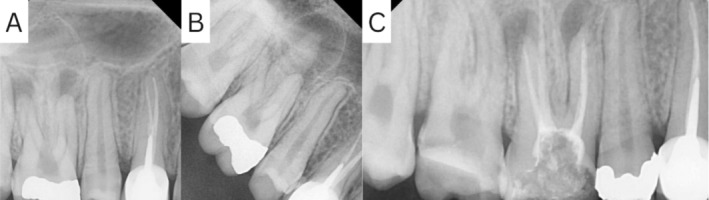
Initial and final radiographs taken by the referring dentist: (A) pre‐operative periapical radiograph; (B) pre‐operative mesial‐angulated radiograph; (C) post‐treatment radiograph after the initial RCT with temporary restoration.

The maxillary right first molar (Universal #3) of a 32‐year‐old woman was evaluated at the Endodontics Clinic of Mashhad Dental School, Iran, for recurrent infection and discharge from the buccal and palatal aspects. She had been referred by a general dentist who examined her 2 weeks prior. In the referral report, the dentist noted signs of infection and a history of pus drainage through the fistula in the past 2 months. Pre‐operative radiographs and clinical photographs of the initial condition and treatment were provided by the referring dentist, as shown in Figure 1.

### 2.2 Methods (Differential Diagnosis, Investigations and Treatment)

Radiological and clinical evaluations indicated pulp necrosis along with chronic apical periodontitis. The patient reported painful sensitivity to touch and percussion, as well as spontaneous pain. Given the history of discharge from both sides of the tooth, vertical root fracture (VRF) and bilateral maxillary sinus tracts were considered in the differential diagnosis, with differing prognoses for each condition. Sinus tract tracing was not possible, because the tract had closed after antibiotic therapy initiated 2 weeks earlier. These antibiotics had been prescribed by the referring dentist following initial Root Canal Therapy (RCT). However, probing depth assessment revealed no J‐shaped lesion, making VRF less likely. Although cone beam computed tomography (CBCT) is the preferred adjunct for evaluating suspected VRF, it was not performed due to the patient's preference to avoid additional radiation and financial constraints. The clinical course and key interventions are summarized in Table 1.

**TABLE 1:** Timeline of events from initial symptoms to 9‐month follow‐up.

Time point	Event	Details
~2 months before presentation	Symptom history	Referring dentist reported ~2 months of pus drainage through the fistula
~2 weeks before presentation	Initial RCT + antibiotics	Patient was examined by a general dentist ~2 weeks earlier; antibiotics were started after the initial RCT, and the tracts temporarily closed (radiographs from referring dentist: Figure 1)
Day 0 (Endodontics clinic visit)	Presentation + diagnostic assessment	Maxillary right first molar involved; bilateral buccal/palatal drainage history. Sinus tract tracing was not possible because the tract had closed after antibiotics. VRF considered; probing did not show a characteristic J‐shaped defect. CBCT was not performed due to financial and radiation‐related constraints (limitations)
Day 0	Intervention	Single‐visit nonsurgical root canal retreatment performed (working length, master cone, obturation: Figure 2A–C)
+48 h	Early outcome (subjective)	Patient reported subjective symptom relief within 48 h
+4 weeks	Follow‐up	Follow‐up radiograph obtained (Figure 3A)
+6 months	Follow‐up	Radiographic healing of apical lesions and clinical improvement documented (Figures 3B and 4B1,B2)
+9 months	Final follow‐up	Complete clinical resolution of both intraoral sinus tracts with no recurrence; radiographic follow‐up shown (Figures 3C and 4C1,C2)

**FIGURE 2:**
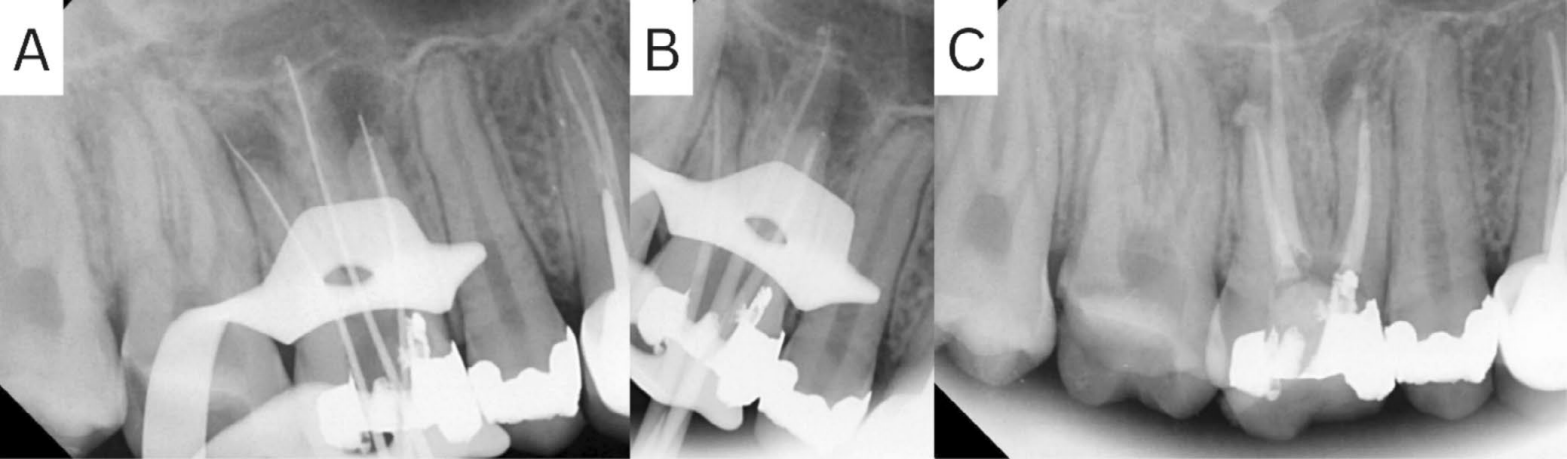
Retreatment radiographs: (A) working‐length confirmation; (B) master‐cone fit confirmation; (C) obturation (final radiograph).

The patient underwent a single‐visit root canal retreatment as the first‐line approach for a potentially savable tooth. The procedure began with the administration of local anesthesia using 2% lidocaine and epinephrine 1:100,000 (Daroupakhsh, Tehran, Iran). The temporary restoration was carefully removed using a high‐speed diamond round bur number 2 (Jota AG, Rüthi, Switzerland) and a continuous water spray. The entire process was performed under rubber dam isolation and with a dental operating microscope (Zumax Medical Co., Suzhou New District, China) to ensure precision and safety. To prepare the root canals, gutta‐percha was removed with chloroform (Morvabon, Tehran, Iran), followed by coronal flaring with Gates‐Glidden drills (#1–3) and re‐instrumentation with M3 retreatment rotary files (UDG, Changzhou, China). The working length of the canals was determined using an electronic apex locator (Dempex, DEM Ltd., England) and verified through radiography (Figure 2A). Root canals were prepared using a crown‐down technique with M3 rotary files (UDG, Changzhou, China) up to size 25/0.04, except for the MB2 canal, which was shaped to a smaller size (size 20/0.04). The MB2 canal was found and classified as Vertucci type II, merging with MB1 in its coronal part. Extensive irrigation and passive ultrasonic activation of sodium hypochlorite and normal saline alternately were performed during canal instrumentation. After confirming the cone fit through radiography (Figure 2B), the canals were dried using sterile paper points (META, South Korea) and obturated with gutta‐percha (META, South Korea) and a bioceramic sealer (NeoSEALER Flo, Avalon Biomed, Houston, TX, USA) using a warm vertical technique (FastFill Obturation System, Eighteeth, China) (Figure 2C).

### 2.3 Outcome and Follow‐Up

**FIGURE 3:**
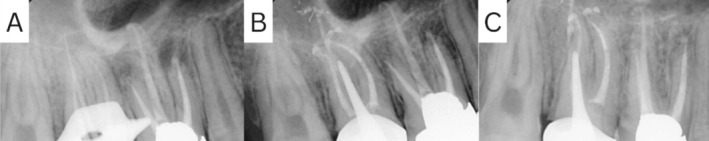
Post‐operative follow‐up radiographs: (A) 4 weeks; (B) 6 months; and (C) 9 months. Note the simultaneous treatment of the upper second molar during the first follow‐up session.

The patient reported subjective symptom relief within 48 h after retreatment. The primary outcomes were radiographic healing and clinical closure of the sinus tracts: a six‐month follow‐up radiograph showed healing of apical lesions, and at 9 months the intraoral sinus tracts had completely resolved with no recurrence. Post‐operative follow‐up radiographs at 4 weeks, 6 months, and 9 months are provided in Figure 3.

**FIGURE 4:**
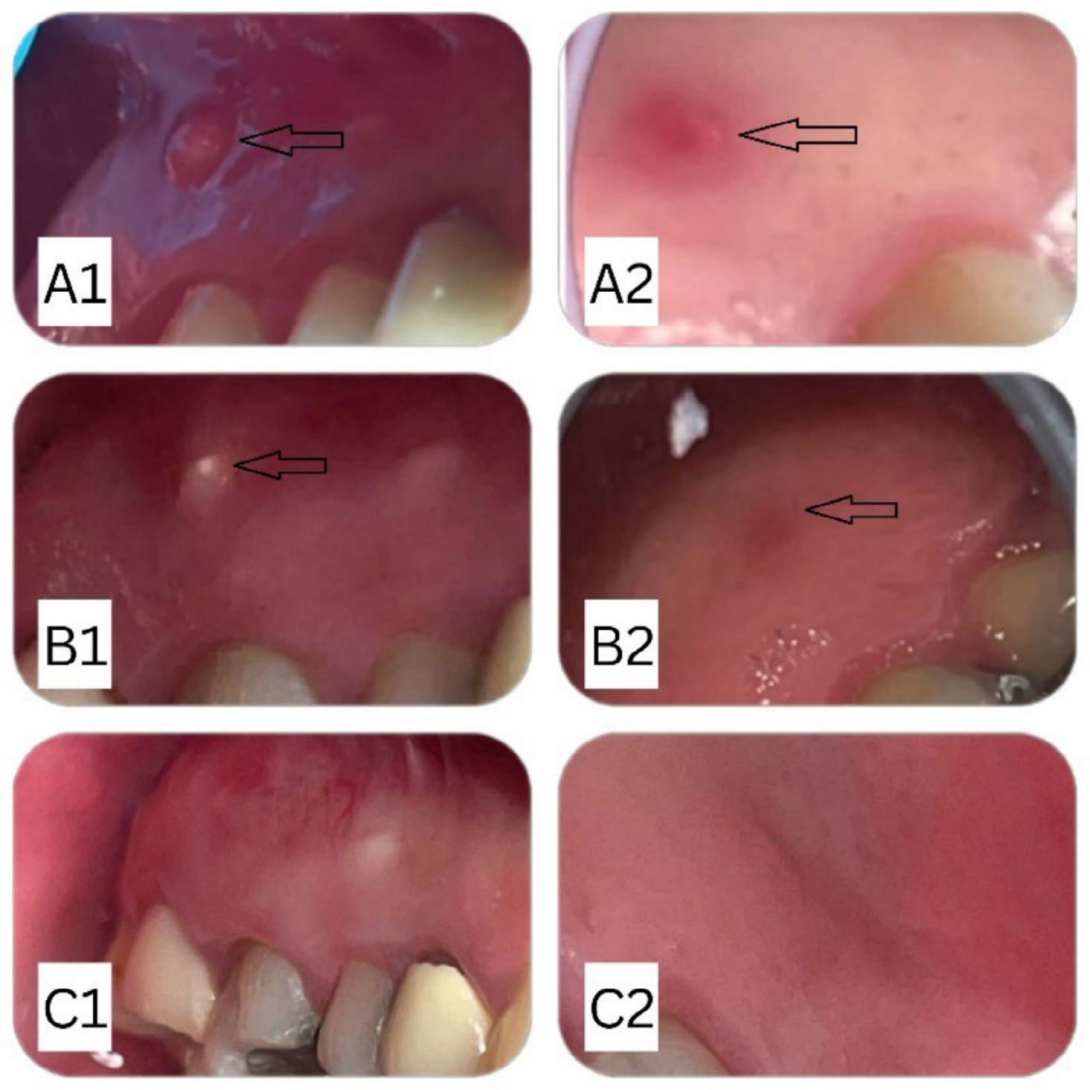
Photographs of clinical appearance on both buccal and palatal sides; (A1) Pre‐operative, buccal view; (A2) Pre‐operative, palatal view; (B1) 6‐month follow‐up, buccal view; (B2) 6‐month follow‐up, palatal view; (C1) 9‐month follow‐up, buccal view; (C2) 9‐month follow‐up, palatal view.

Photographs taken from the buccal and palatal views in the pre‐operative session and at the 6‐ and 9‐month follow‐ups also showed signs of healing of the fistulas (Figure 4).

## 3 Discussion

Considering the proximity of the apices to the lingual or palatal plate in mandibular and maxillary molars, the presence of lingual or palatal fistula is more likely than buccal tracts [9]. A single fistula is typically observed; however, bilateral sinus tracts are commonly associated with vertical root fractures [10]. The case presented here highlights the importance of thorough diagnostic evaluation and the potential for unusual presentations in dental practice.

A correct diagnosis is often considered three‐fourths of the remedy, emphasizing the importance of accurately determining the origin of a lesion (odontogenic or non‐odontogenic) for effective management. Patients with odontogenic lesions in atypical locations may undergo numerous surgical excisions, radiotherapy sessions, multiple biopsies, and various antibiotic treatments. However, since these treatments do not address the odontogenic origin of the lesion, the sinus tract frequently recurs [11].

Previous studies highlighted the need for timely intervention and the benefits of using imaging techniques for accurate diagnosis [12, 13]. CBCT is particularly useful in diagnosing complex endodontic issues, especially when VRF is suspected. It provides a three‐dimensional view of periapical lesions and surrounding anatomical structures [14]. However, when CBCT is limited by cost, access, or patient preference regarding radiation, careful clinical assessment remains an acceptable basis for conservative management. In the present case, CBCT was not performed owing to a clear periapical diagnosis after failed primary RCT, the patient's refusal of additional radiation, and financial constraints. Nevertheless, clinical evaluation of probing depth helped rule out the VRF diagnosis.

A recent review of the maxillary sinus underscores that its anatomy—and the intimate proximity of posterior maxillary roots—drives a spectrum of odontogenic sinonasal disease and endo‐antral communications [15]. Within this framework, careful endodontic evaluation remains central to identifying dental sources of sinonasal pathology. Consistent with these insights, the bilateral tracts in our patient are most plausibly odontogenic in origin despite the absence of vertical root fracture.

Once a dental origin is correctly diagnosed, several differential diagnoses should be considered, including tumors [16], cysts [17], or a simple chronic dental infection. Treatment for odontogenic lesions can range from non‐surgical approaches to more advanced surgical procedures. When a sinus tract persists, the well‐epithelialized cord‐like tissue often hinders healing through conventional endodontic treatment by preventing complete disinfection, allowing bacteria to persist in the periapical lesion. In cases of chronic odontogenic sinus tracts, root canal treatment alone may be insufficient, requiring the removal of the cord‐like tract from the alveolar bone or its complete excision through surgery like apicoectomy [18, 19].

This case report highlights the value of thorough diagnostic evaluations. The nine‐month follow‐up period provides valuable insights of sustained healing and management of such conditions. However, the absence of CBCT, the inability to perform sinus tract tracing, and a single‐case basis limit generalizability. Further research with larger sample sizes is needed to confirm these findings and develop standardized guidelines.

## 4 Conclusion

This case report highlights the importance of diagnosing bilateral sinus tracts of dental origin, noting that they do not necessarily indicate VRF in all cases. Endodontic treatment is generally effective in managing these tracts and preventing recurrence. While endodontic surgical intervention may sometimes be required, it is typically not the first‐line treatment. It is recommended to prioritize a dental‐first evaluation and conservative endodontic retreatment for suspected odontogenic sinus tracts in daily practice, with adequate follow‐up (6–9 months) to confirm healing and preserve the tooth when possible.

## Author Contributions

**Ali Chamani:** conceptualization, methodology, project administration. **Maryam Forghani:** investigation, supervision, writing – review and editing. **Reza Shakiba:** writing – original draft, writing – review and editing. **Arsalan Shahri:** data curation, writing – original draft.

## Funding

Self‐funded.

## Consent

Written and verbal informed consent was obtained from the patient to report this case and accompanying images.

## Conflicts of Interest

The authors declare no conflicts of interest.

## Data Availability

The data supporting this study's findings are available upon reasonable request from the corresponding author. The data are not publicly available due to privacy and ethical restrictions.
